# Does the Wim Hof Method have a beneficial impact on physiological and psychological outcomes in healthy and non-healthy participants? A systematic review

**DOI:** 10.1371/journal.pone.0286933

**Published:** 2024-03-13

**Authors:** Omar Almahayni, Lucy Hammond

**Affiliations:** Warwick Medical School, University of Warwick, Coventry, United Kingdom; Hamasaki Clinic, JAPAN

## Abstract

**Introduction:**

Wim Hof, also known as the iceman, developed a method called Wim Hof Method (WHM) which he claims to have several benefits on physical and mental health. The aim of this systematic review is to identify and synthesise the results of the studies conducted on WHM on physiological and psychological health-related outcomes.

**Materials and methods:**

This systematic review followed the PRISMA guidelines for systematic reviews. The protocol was registered in the International Prospective Register of Systematic Reviews (PROSPERO), registration number CRD42022333209. Medline and Web of Science were searched and included studies from January 1, 2014, to July 4, 2022. Studies were included if they met the predetermined inclusion/exclusion criteria. Inclusion criteria included RCTs and cohort studies published in peer-reviewed journals, studies conducted on healthy individuals and people with pre-existing medical conditions (adolescents and adults over the age of 14), studies that included all three pillars (breathing, cold exposure, and commitment) of the WHM, and studies that only focused on Wim Hof breathing method (WHBM). Exclusion criteria included studies that discussed WHM but are not original experimental research or are not peer-reviewed, studies that included children under the age of 14, and studies that used methods similar to WHM, but not actually WHM, such as tummo meditation. The articles were assessed for risk of bias using RoB 2.0 and Scottish Intercollegiate Guidelines Network (SIGN) tools. The effects of WHM were categorised into physiological or psychological related outcomes and narrative synthesis was conducted.

**Results:**

Nine papers were included in this review which consisted of eight individual trials. The findings suggest that the WHM may reduce inflammation in healthy and non-healthy participants as it increases epinephrine levels, causing an increase in interleukin-10 and a decrease in pro-inflammatory cytokines. Additionally, effect of WHBM on exercise performance showed mixed findings. Effects on respiratory parameters of minute ventilation, tidal volume, and breathing frequency were mixed following bouts of exercise.

**Conclusion:**

Taken together, the findings of this review show promising use of WHM in the inflammatory response category. The focus of future studies should further investigate the benefits of WHM in non-healthy participants with inflammatory disorders and explore the use of Wim Hof breathing method to enhance exercise performance.

## Introduction

The Wim Hof Method (WHM) is a lifestyle change that claims to enhance a person’s quality of life which consists of three pillars: Wim Hof breathing method (WHBM), cold therapy, and commitment. According to Wim Hof, the creator of this method, a combination of these three pillars enhances a person’s autonomic nervous and immune systems, thus strengthening a person’s health and mentality.

Wim Hof is recognized for his ability to resist extreme cold temperatures. He holds 21 Guinness World Records for some of his remarkable human achievements, including climbing Mount Kilimanjaro while wearing shorts, swimming 66 metres beneath ice, standing for two hours in a container filled with ice cubes, and running a half marathon over the Arctic Circle, only wearing shorts and barefoot [1]. Wim Hof credits his achievements to the WHM.

The first pillar of the WHM is WHBM which consists of hyperventilation 30–40 times then holding the breath voluntarily at low lung volume [2]. Secondly, cold therapy involves taking daily cold showers or sitting in an ice bath [3]. The final pillar, which is commitment, is the foundation of the other two pillars; mastering both mindful breathing and cold exposure takes patience and persistence [4]. To support his claims, Wim Hof has submitted his method and himself for research. Many studies, including case studies, randomised control trials (RCTs), and observational trials have been conducted on him and his method [5–16].

To identify some of the benefits people are experiencing, Wim Hof collaborated with RMIT University by conducting a survey inquiring about the impact WHM had on an individual’s health and well-being [17]. Over 3,200 people answered the survey, and the majority of the answers were positive. Respondents reported a good mix of physical and mental benefits such as an increase in their tolerance to resist cold, as well as an increase in energy, mood, mental focus, and general health. The survey findings also claimed that the WHM had benefits for specific conditions such as stress, tiredness and fatigue, anxiety, depression, back pain, insomnia, arthritis, and chronic pain [17].

Collectively, these findings suggest that the WHM may be of importance to healthy and non-healthy individuals and that the WHM approach may be given consideration in the public health and lifestyle medicine fields. The notion of lifestyle medicine refers to the study of how actions and habits affect illness prevention and treatment [18]. However, caution should be applied insofar as the aforementioned survey [17] has not been peer-reviewed or published, and Wim Hof’s own involvement in this study and others [5, 9] may give rise to conflicts of interest in the pursuit of both an evidence base and a commercial opportunity. Wim Hof has attained a level of celebrity as a result of his achievements, method and associated media career. Due to all his remarkable achievements, his charisma, and authenticity, Wim Hof might be considered to be both achieved and micro celebrity [19], where achieved celebrity refers to the attainment of fame based on recognized talent, achievement, or ability and micro celebrity describes an individual that achieves status and social media presence by self-broadcasting about niche subjects to a small community of followers. Wim Hof is an example of a celebrity that embodies the para-social relationship, exchanging the allure of intimacy, integrity, and authenticity. The growing number of celebrities giving lifestyle and health advice and scientific knowledge to the public indicates the need to critically examine such advice [20]. Nunan *et al*. urge caution for the potential of “the unintended consequences of uncritical endorsement and application of lifestyle medicine including the infiltration of pseudoscience, profiteering, and the potential for widening health inequalities by a continued focus on the ‘individual’” (p229) [21]. Therefore, an independent, systematic synthesis of this evidence is warranted to evaluate the positive health claims of the WHM.

A systematic review has not yet been conducted on the WHM body of evidence. Therefore, an independent, systematic synthesis of this evidence is warranted to evaluate the positive health claims of the WHM. Hence, this review aims to identify and synthesise the results of the studies conducted on WHM on physiological and psychological health-related outcomes.

## Materials and methods

This systematic review followed the PRISMA guidelines for systematic reviews [22]. The protocol was registered in the International Prospective Register of Systematic Reviews (PROSPERO), registration number CRD42022333209.

### Literature search

MEDLINE and Web of Science databases were used to search for published studies for potential inclusion in this systematic review. The following search phrases were used in combination: Wim Hof; breathing exercise OR breathing technique; meditation OR commitment; cold temperature OR cold exposure OR ice bath OR cold shower. The filters English language, year = "2014—July 4, 2022", and journal articles or observational studies or randomised controlled trials (RCTs) were applied. A filter for the year was applied because the first study performed on WHM was in 2014 [10].

### Study selection

Duplicates were deleted before abstracts were screened. The screening of titles, abstracts and full texts was conducted by two independent reviewers, with any disagreements resolved by consensus. Screening was performed in Covidence (title and abstract screening, full text review, and extraction of the chosen articles). Studies were included according to the predetermined inclusion/exclusion criteria. Inclusion criteria included RCTs and cohort studies published in peer-reviewed journals, studies conducted on healthy individuals and people with pre-existing medical conditions (adolescents and adults over the age of 14), studies that included all three pillars (breathing, cold exposure, and commitment) of the WHM as defined above, and studies that only focused on WHBM. Articles just covering WHBM were permitted as it has been deemed more important and studied more frequently than other pillars. Furthermore, exclusion criteria included studies that discussed WHM but are not original experimental research or are not peer-reviewed, studies that included children under the age of 14, and studies that used methods similar to WHM, but not actually WHM, such as Tummo meditation. Both WHM and Tummo methods allow for the controlment of body temperature, and both have similar breathing techniques, however Tummo meditation consists of breathing and visualisation techniques which are intended to summon spiritual insight, whereas WHM is not religious [23]. After deciding on the included studies, the reference lists were hand-searched for other relevant articles.

Data extraction comprised the name of the study, place where the study was conducted, research design, participant demographics, study context, description of intervention and control, and key findings. One reviewer completed the extraction while a second reviewer checked it for accuracy. Furthermore, study quality was assessed using the RoB 2.0 risk bias tool [24] for RCTs and the Scottish Intercollegiate Guidelines Network (SIGN) risk bias tool [25] for cohort studies. Two reviewers independently evaluated the possibility of bias. The answers were then compared, and any disagreement was discussed between the reviewers.

### Data synthesis

A narrative synthesis of the findings from the studies was included by providing a description and comparison between the different studies. This comprised a discussion of the contexts in which the studies have been undertaken, and the effects of WHM thematically grouped into physiological or psychological related outcomes. In each thematic grouping, different sets of outcomes were included. The data were not suitable for meta-analysis as there were various unrelated outcome measures resulting in heterogeneity across studies.

## Results

### Study characteristics

**Fig 1:**
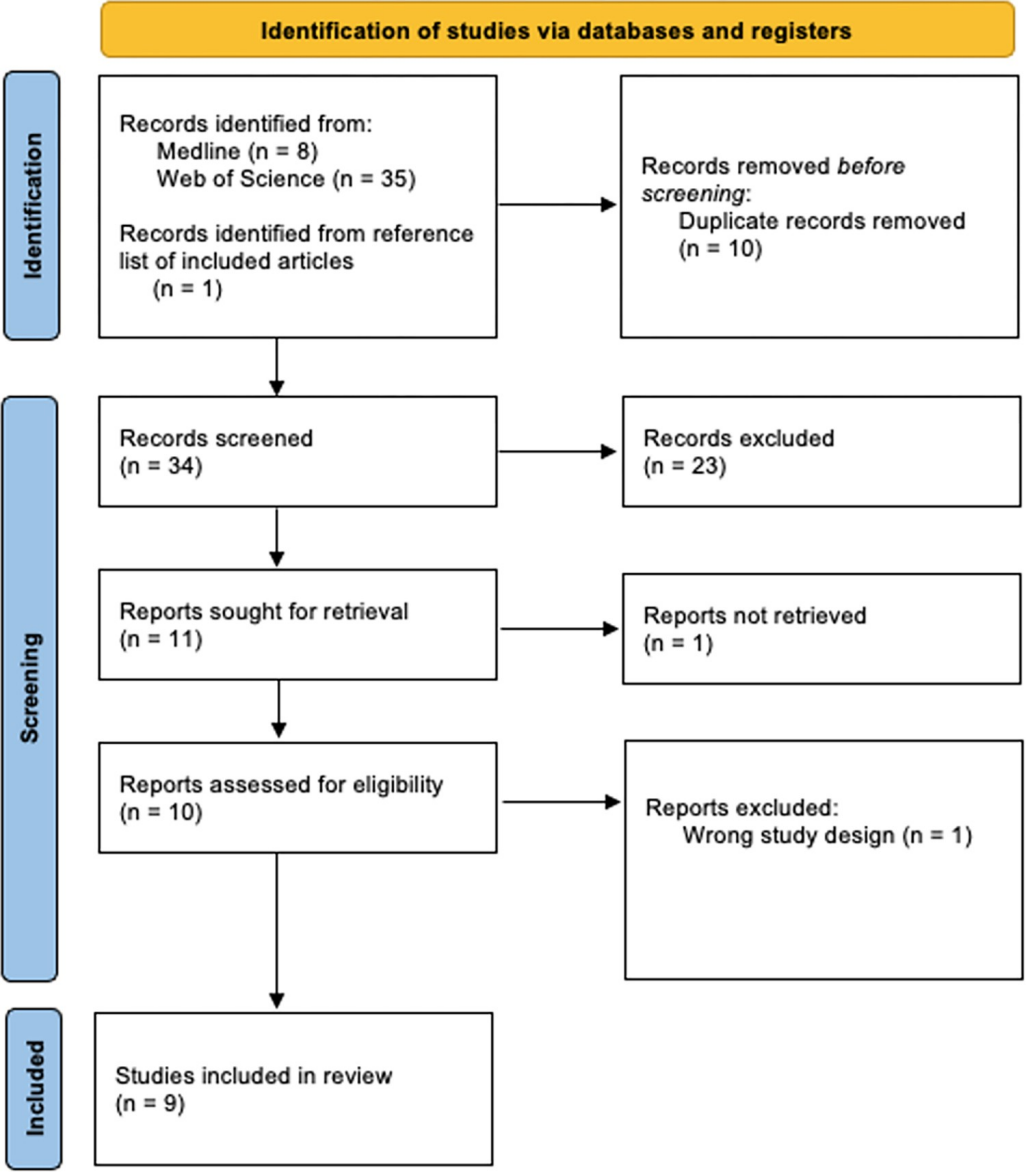
PRISMA flow diagram for study selection processes.

Nine papers, seen in Fig 1, published between 2014 and July 4, 2022, were identified that examined the WHM. One of these articles was identified from hand-searching the included studies’ reference lists [6]. Two of these articles [13, 16] were further analyses of a study [10]. Table 1 summarises the characteristics of the listed studies. Of the nine papers included, there were eight individual trials. Four of the trials performed the full WHM [7, 10, 12, 15] and four did only WHBM [6, 8, 11, 15]. The application of WHBM differed between studies as some studies had one breathing exercise [6, 8, 11, 12] while others had two breathing exercises [7, 10, 13, 15, 16]. The first breathing exercise was common in all studies. Cold exposure was conducted according to what was accessible to each study. However, all studies agreed on daily exposure to the cold through having cold showers, but the duration of exposure under the shower differed across studies. The meditation technique was the least focused component of the WHM. All studies had the same description of the meditation technique except for Petraskova Touskova *et al*. [12]. All the authors described it as visualisations meant to promote complete relaxation, while Petraskova Touskova *et al*. described it as a form of focusing to obtain self-awareness and will-power. Out of the nine papers, five conducted a randomised controlled trial (RCT) design [10, 11, 13, 15, 16], three conducted a crossover RCT design [6–8], and one conducted a prospective cohort design [12].

**Table 1:** Characteristics of the listed studies.

Study	Country	Study Design	Population Description	WHM Intervention	Description of time over which WHM method practised	Control’s Group Intervention	Additional Experimental Exposure
Kox *et al*., 2014	Netherlands	RCT Evaluating effect of WHM on symptoms following endotoxemia administration.	n = 24 30 were recruited, but only 24 completed the full protocol. Sex: male. Age range: 19–27 years. Status: healthy (normal electrocardiography, physical examination, and routine laboratory values). Non-smoking.	The study was performed in two identical blocks, consisting of nine participants in the WHM-trained group in each block. Six participated in the endotoxemia experiments and six subjects in the control group. The WHM-trained group was trained by three trainers and Wim Hof. The day before the endotoxemia experiment, Wim Hof held a last training session in which the chosen participants practised WHM.	10 days of training. The first 4 days took place in Poland and were most intensive. The individuals performed the methods every day by themselves at home (2–3 hours each day) after returning from Poland until the endotoxemia experiment day (5–9 days later). Cold showers were used for cold exposure. The day before the endotoxemia trial, Wim Hof held a final training session.	No WHM training.	**Endotoxemia administration:** Intravenous administration (iv) administration of 2 ng/kg Escherichia coli endotoxin.
Middendorp *et al*., 2016	Netherlands	RCT Evaluating the role of the participants outcome expectancies of WHM following endotoxemia administration.	Same as Kox *et al*., 2014	Same as Kox *et al*., 2014.	Same as Kox *et al*., 2014.	Same as Kox *et al*., 2014.	Same as Kox *et al*., 2014.
Buijze *et al*., 2019	Netherlands	Open label randomised one-way cross over design. Evaluating WHM effect on anti-inflammatory response in axial spondyloarthritis patients.	n = 19 24 were recruited, but only 19 completed the full protocol. Sex: males and females. Age range: 18–55 years. Medical status: clinical diagnosis of axial spondyloarthritis. Medication use: non-steroidal anti-inflammatory drugs, corticosteroids, or disease modifying anti-rheumatic drugs.	**Early intervention** Participants were trained by four trainers and Wim Hof. **Late intervention** The participants in the control group also received exposure to the intervention after the 8-week control phase had ended. Patients were trained in the same way as the early intervention.	8-weeks add-on training program (4 weeks of training twice weekly then 4 weeks of training once weekly).	8 weeks of control period during the early intervention. No WHM training.	N/A
Zwaag *et al*., 2020	Netherlands	RCT Evaluating WHM effect on the anti-inflammatory responses of lactate and pyruvate.	Same as Kox *et al*., 2014.	Same as Kox *et al*., 2014.	Same as Kox *et al*., 2014.	Same as Kox *et al*., 2014.	Same as Kox *et al*., 2014.
Bahenský *et al*., 2020	Czech Republic	Crossover RCT Evaluating WHBM effect on oxygen consumption (VO_2_) in young, endurance trained individuals.	n = 16 Sex: males and females. Age range: 16–19 years. Trained: medium and long-distance runners. Running six times every week for at least three years.	Breathing exercises were conducted while lying down.	Single WHBM session. The first group completed the cycle ergometer testing sessions without and then with WHBM, whereas the second group reversed the order.	Rest quietly while sitting down.	**Exercise test:** All participants undertook an incremental cycle ergometer testing session that compromised: warm-up (2 min at 25 W), followed by four two-minute stages of increasing intensity (1, 2, 3, 4 W/kg) based on the body mass of each participant.
Citherlet *et al*., 2021	Switzerland	Randomised, controlled three-way crossover trial. Evaluating WHBM effect on repeated sprint ability.	n = 15 16 participants enrolled, but 1 was excluded during the trial. Sex: male. Age range: ≥ 18 years. Status: healthy regular runner.	Practised WHBM.	Only once by each participant.	Spontaneous breathing for 12 min. Hyperventilation.	N/A
Zwaag *et al*., 2022	Netherlands	RCT Evaluating effect of cold exposure and WHBM (with or without prolonged breath retention) on inflammatory response.	Sex: males. Medical status: healthy. **Breathing exercise study** n = 40 Age range: 19–24 years **Endotoxemia study** n = 48 Age range: 20–24 years	**Breathing exercises study** Extensive or short training by Wim Hof. Extensive or short training by an independent trainer. **Endotoxemia study** Cold exposure alone (CEX). Breathing exercise without retention alone (BRT). Cold exposure and the breathing exercise without retention (CBR).	**Breathing exercises study** Participants who received the extensive training by the independent trainer or Wim Hof, were trained for 4 days (every morning for 2 hours). Participants were then told to practise the learned exercises at home, after finishing the 4 days. Participants who received the short training by the independent trainer or Wim Hof, were trained only on the fourth day (2 hours on the morning of the fourth day). Participants were told not to practise at home. **Endotoxemia study** The CEX group followed an intensive 4-day cold exposure training programme. Participants were told to take a 1 minute cold water rinse at the end of each day’s shower. The BRT group had 2 hours of training with an independent trainer. Participants were told not to practise at home.	**Breathing exercises study** No control group. **Endotoxemia study** No WHM training.	**Endotoxemia exposure:** Purified lipopolysaccharide (LPS) was reconstituted in 5 mL of saline 0.9% for injection, vortexed for 20 minutes, and then infused as an iv bolus at a dose of 2 ng/kg body weight for 1 minute at time t = 0 hours at 9:30AM.
Petraskova Touskova *et al*., 2022	Czech Republic	Prospective observational study. Evaluating effect of WHM on hormone release and stress response.	n = 13 Sex: females and males. Age range: 27–60 years. Status: all had American Society of Anesthesiology health scale scores of 1 to 2. One male participant was a mild smoker. The other participants were non-smokers.	Participants practised WHM.	The intervention group attended WHM training led by a certified instructor about a month before the excursion. The intervention consisted of 8 weeks of WHM training.	No WHM training.	N/A
Marko *et al*., 2022	Czech Republic	RCT Evaluating effect of WHBM on breathing economy during exercise.	N = 19 Sex: males and females. Age range: 14–19 years. Status: healthy (free from known metabolic, cardiovascular, and/or renal disease). Trained: middle and long-distance runners.	Performed WHBM for 17–22 mins.	4 week interventional program. Conducting breathing technique with an empty stomach on daily basis.	Breathe naturally while in supine position.	**Exercise test:** The test and follow-up test involved a breath-by-breath metabolic study after a graded exercise test (GXT) on a bicycle ergometer. Participants finished four, two-minute stages at 1, 2, 3, and 4 W/kg after a preliminary warm-up at 25 W and a cadence of 80 rev/min. Throughout the test, participants were told to keep their cadence at around 100 revs/min. A three-minute relaxation with a cadence of 60 rev/min and resistance of 25 W took place after the final workload phase (4 W/kg).

### Study quality

For the RCT studies, the RoB 2.0 overall risk of bias judgement was ‘high concerns’ for all studies except one study which was judged as ‘some concerns’ [8] (Table 2). Most of the articles were judged as high concern due to the difficulty in blinding the participants and researchers to the intervention. For the cohort study [12], the SIGN tool was used. The cohort study was scored ‘unacceptable’ as it was not able to reduce the possibility of bias or confounding. An explanation of the results of the SIGN tool can be found in the S2 Table.

**Table 2:** RoB 2.0 risk of bias domians [26].

	Domain 1: Randomisation process	Domain 2: Alterations from planned interventions	Domain 3: Missing outocme data	Domain 4: Outcome measurement	Domain 5: Reported results that were selected	Overall
Kox *et al*., 2014	Low concerns	High concerns	Low concerns	Low concerns	Some concerns	High concerns
Middendorp *et al*., 2016	Low concerns	High concerns	Low concerns	High concerns	Some concerns	High concerns
Buijze *et al*., 2019	Low concerns	High concerns	Low concerns	Low concerns	Some concerns	High concerns
Zwaag *et al*., 2020	Low concerns	High concerns	Low concerns	Low concerns	Some concerns	High concerns
Bahenský *et al*., 2020	Some concerns	Some concerns	High concerns	Low concerns	Some concerns	High concerns
Citherlet *et al*., 2021	Some concerns	Some concerns	Low concerns	Low concerns	Some concerns	Some concerns
Zwaag *et al*., 2022	Low concerns	High concerns	Low concerns	High concerns	Some concerns	High concerns
Marko *et al*., 2022	Low concerns	High concerns	High concerns	Low concerns	Some concerns	High concerns

### Outcomes

Table 3 summarises the WHM description, key findings, and quality assessment. The outcomes were separated into physiological or psychological outcomes. The physiological outcome has six categories: stress response, pro-inflammatory/anti-inflammatory response, metabolites response, respiratory parameters, blood gas measurements, and reporting of symptoms, while psychological outcome has one category: psychological response. The stress response included outcomes such as epinephrine, norepinephrine, dopamine, cortisol, and heart rate (HR) whereas pro-inflammatory/anti-inflammatory response included tumour necrosis factor (TNF-α), interleukin-6 (IL-6), interleukin-8 (IL-8), interleukin-10 (IL-10), erythrocyte sedimentation rate (ESR), Ankylosing Spondylitis Disease Activity Score C-reactive protein (ASDAS-CRP), calprotectin, high-sensitivity C-reactive protein (hs-CRP), and Bath Ankylosing Spondylitis Disease Activity Index (BASDAI). Additionally, metabolites response contained lactate and pyruvate while respiratory parameters looked at minute ventilation (V_E_), tidal volume (V_T_), and breathing frequency (BF). The blood gas measurements category included carbon dioxide partial pressure (pCO_2_), pH, and oxygen saturation outcomes and the psychological response category contained expectancy, optimism, neuroticism, short-form 36 (SF-36), EuroQol-5D (EQ-5D), Borg Rating of Perceived Exertion (RPE) scale, and customised questionnaire. Finally, the reporting of symptoms investigated flu-like, self-reported, and depressive symptoms, and Trauma Symptom Checklist-40 (TSC-40).

**Table 3:** Summary of WHM description, key findings, and quality assessment.

Study	Description of meditation component of WHM	Description of breathing technique of WHM	Description of cold exposure of WHM	Key findings
Kox *et al*., 2014	Third eye meditation is a type of meditation that includes visualisations aimed at complete relaxation.	Two breathing exercises. The first exercise is the same as Bahenský *et al*., 2020. The second exercise consisted of deep inhalations and exhalations. After each inhalation and exhalation breath was held for 10s, during which the individual tightened all the muscles. The breathing techniques were also implemented during endotoxemia. Strength workouts were also included in the training programme (e.g., yoga balance techniques and push-ups).	Standing in the snow barefoot for up to 30 minutes. Lying bare chested in the snow for 20 minutes. Daily swimming or dipping in ice-cold water for several minutes. Hiking a snowy mountain (5–12°C) bare chested, wearing nothing but shoes and shorts.	Intermittent hypoxia and respiratory alkalosis brought on by WHM markedly raised epinephrine levels. After endotoxin treatment, IL-10 levels increased more quickly and were higher. They also showed a substantial correlation with baseline epinephrine levels. TNF-α, IL-6, and IL-8 levels were lower in WHM-trained group and correlated negatively with IL-10 levels. Flu-like symptoms were lower in the WHM-trained group.
Middendorp *et al*., 2016	Same as Kox *et al*., 2014.	Same as Kox *et al*., 2014.	Same as Kox *et al*., 2014.	Generalised outcome expectancy optimism is a potential predictor of the immune and autonomic response to induced inflammation following training, while more specific expectations regarding the training’s effects might be especially relevant for the clinical symptom report.
Buijze *et al*., 2019	Same as Kox *et al*., 2014.	Same as Kox *et al*., 2014.	Patients submerged their entire body for several minutes in ice-cold water (0–1°C) incrementally up to a maximum of 5 minutes. Cold showers were taken incrementally up to 5 minutes (10–14°C) daily at home.	No significant differences in adverse events between groups, with one serious adverse event occurring 8 weeks after finishing the intervention and judged ‘unrelated’. During the 8-week intervention period, there was a significant decline of erythrocyte sedimentation rate (ESR), Ankylosing Spondylitis Disease Activity Score C-reactive protein (ASDAS-CRP), and serum calprotectin, whereas no effect was found in the control group. ESR, ASDAS-CRP, and high-sensitive CRP (hs-CRP) are endpoints that measure inflammation. High ESR, ASDAS-CRP, or hs-CRP means there is inflammation. The decline in ESR, ASDAS-CRP, and serum calprotectin suggest that there is a decrease in inflammation. No significant decline was found for hs-CRP.
Zwaag *et al*., 2020	Same as Kox *et al*., 2014	Same as Kox *et al*., 2014.	Same as Kox *et al*., 2014.	The training program’s effects were most noticeable soon after WHBM started but before LPS was administered, and they point to greater activation of the Cori cycle. Increased levels of IL-10 were associated with elevated lactate and pyruvate concentrations in trained individuals. Co-incubation with lactate and pyruvate increases IL-10 production while decreasing the release of pro-inflammatory IL-1 and IL-6 by LPS-stimulated leukocytes, according to in vitro validation trials.
Bahenský *et al*., 2020	N/A	Participants were instructed to take 30–50 complete breaths at a pace of 20 breaths/min. Exhale completely when tingling in the fingers is noticed. The participants were told to hold their breath until they felt a strong urge to breathe or until the diaphragm spontaneously contracted. Each breath hold was followed immediately by a complete breath then a secondary 15-sec breath hold. Three similar rounds were performed without rest, lasting between 17–22 min.	N/A	During the testing stages, preceded by the breathing techniques, O_2_ consumption was significantly higher (2.4–4.9%; p<0.05). Following breathing exercises, perceived effort was reduced throughout the exercise session (18.5±1.2 vs. 17.4±1.1; p<0.01). Prior to an acute exercise bout, breathing techniques including deep breathing and breath holds appear to enhance VO2 kinetics and minimise subjective strain in participants.
Citherlet *et al*., 2021	N/A	Participants followed the audio guide on the WHM application. WHBM was repeated for three cycles. One cycle consisted of 30 breaths of hyperventilation, defined as maximal amplitude respiratory movements at the frequency specified by the audio guidance (0.32 Hz). This process lasted for approximately 1 minute and 30 seconds. The participants then exhaled completely to residual volume and kept their breath for as long as they could. Afterwards, the participants inhaled to total lung capacity and held their breath for 15 seconds before beginning the next cycle.	N/A	No significant negative or positive condition effects were discovered on Repeated Ability Sprint Test (RAST) peak power, average power, or fatigue index despite considerable physiological effects on the finger pulse oxygen saturation (SpO_2_) and expired carbon dioxide (VCO_2_) levels of both WHBM and hyperventilation (HV). Finger SpO_2_ was reduced at the end of the voluntary breath-holds (BH). End-tidal CO_2_ partial pressure measurements during the final HV in the WHBM and HV conditions were suggestive of respiratory alkalosis with an elevation in arterial pH. After RAST, the 8min cumulated VCO_2_ volumes in the HV and WHBM were higher than in the spontaneous breathing (SB), indicating the depletion of residual CO_2_ stores. 33% of individuals in the WHBM reported deafness and heaviness. For the HV condition, 73% of the individuals reported benefits including decreased fatigue and better breathing. For the WHBM condition, 87% of the participants reported benefits like less fatigue, better breathing, and more energy. In contrast to 53% who said they would not use the WHM in the future, 47% said they intended to employ the WHBM in their future personal training and competition practise. Participants conducting the trial in the WHBM condition felt the most advantaged, followed by the HV condition. WHBM condition resulted in feeling significantly more advantaged than SB condition.
Zwaag *et al*., 2022	N/A	The first exercise is the same as Bahenský *et al*., 2020. In the exercise without retention of breath, participants hyperventilated 30 times using powerful and deep breaths. Participants held their breath for only 10 seconds, during which all muscles were tightened. Afterwards, a new cycle of hyperventilation was started.	Same as Kox *et al*., 2014. Participants were told to take a 1 minute cold water rinse at the end of each day’s shower.	Epinephrine levels increased after performing both breathing exercises, and this effect was independent of training duration or instructor. The breathing exercise raised epinephrine levels in the endotoxemia trial. The LPS-induced inflammatory response was not modulated by cold exposure training alone. The breathing exercise resulted in significantly increased levels of anti-inflammatory and decreased levels of pro-inflammatory cytokines. Training in cold exposure considerably improved the breathing exercise’s immunomodulatory effects. In healthy young males, the in vivo inflammatory response is most effectively attenuated by the combination of cold exposure training and breathing exercise.
Petraskova Touskova *et al*., 2022	Participants were told to focus on‚ body awareness, comprising enhancement of self-awareness and will-power.	Same as Bahenský *et al*., 2020.	Cold exposures were carried out on an individual basis by submersion in the water of Prince Gustav Channel or in the shower. Prior to entering the cold water, participants took five to ten long, deep breaths while contracting particular abdominal and pelvic muscles. Participants were asked to carry out the exercises once per day on their own after the first three supervised sessions. One session was around 20 minutes.	Depressive symptoms decreased over an 8-week training programme. There was a significant reduction in stress response. Cortisol reduction was also observed to be non-significant.
Marko *et al*., 2022	N/A	Same as Bahenský *et al*., 2020.	N/A	There were no significant variations in minute ventilation, tidal volume, or breathing frequency before or after the intervention between the experimental and control groups. Following the 4 week intervention period, there was a non-significant small-to-large effect for an increase in BF and V_E_ in both groups, probably as a result of a decrease in training volume and intensity brought on by the break between competing seasons.

### Stress response

Two trials investigated epinephrine levels during the endotoxemia experiment [10, 15] and one trial studied epinephrine levels during WHBM [15]. Baseline epinephrine levels in WHM-trained participants were significantly higher compared to non-trained participants (p = 0.007). After starting WHBM, epinephrine levels increased further in this group and peaked just before endotoxemia administration and remained elevated until WHBM was stopped [10]. Additionally, in Zwaag *et al*. endotoxemia study [15], participants had no variations in epinephrine levels throughout the experiment (p = 0.48) in both participants exposed to cold (CEX) and participants not performing the WHM (CON). However, an increase in epinephrine levels began much earlier and was significantly greater in both the cold exposure with breathing exercise without retention (CBR) (p = 0.01) and breathing exercise without retention (BRT) (p = 0.04) compared to the CEX and CON groups [15]. Furthermore, in Zwaag *et al*. breathing exercises study [15], both breathing exercises (WHBM with/without retention) raised epinephrine levels, which were unaffected by training duration (short vs extensive training: p = 0.71) or the trainer (independent trainer vs Wim Hof: p = 0.46). However, epinephrine was slightly more sustained in participants performing WHBM with retention compared to without retention (p = 0.003) [15]. Moreover, Kox *et al*. [10] explored norepinephrine and dopamine levels during the endotoxemia trial and found them to be within the reference range throughout the experiment [10].

The associations of cortisol with WHM was also explored. In Petraskova Touskova *et al*. study [12], the group not practising WHM had higher, but statistically non-significant cortisol levels at the end of the expedition (p = 0.327) compared to WHM-trained participants [12]. Whereas Kox *et al*. [10] found no variations in serum cortisol levels between the WHM-trained and non-trained groups. The serum cortisol levels were drawn before and throughout the period of WHM training. The only variation between the two groups was that serum cortisol levels normalised faster in WHM-trained participants [10]. The difference in the results could be due to the different collection procedures for cortisol. Kox *et al*. [10] collected cortisol from blood while Petraskova Touskova *et al*. [12] collected hair cortisol.

Four trials examined HR recordings [6, 8, 10, 15]. Kox *et al*. [10] showed an increase in HR after starting WHBM which normalised faster in WHM-trained group compared to non-trained group [10]. Furthermore, Bahenský *et al*. [6] reported that HR tended to be lower after WHBM compared to no WHBM [6]. Citherlet *et al*. [8] also found HR significantly increased during hyperventilation (HV) and significantly decreased during breath hold in participants practising WHBM when compared to resting values [8]. Similarly, Zwaag *et al*. [15] observed a significant increase in HR in both CBR (p<0.001) and BRT (p<0.001) groups, during the first hyperventilation cycle, compared to the CON group. No changes in HR responses between CEX and CON groups (p = 0.89) were found during the endotoxemia experiment. The HR measurements of BRT and CBR groups were comparable to those of CEX and CON groups when WHBM was stopped [15]. In conclusion, HR was increased when WHBM was initiated and decreased when WHBM was stopped compared to control group.

### Pro-inflammatory/anti-inflammatory responses

Two trials investigated cytokines levels during endotoxemia experiment [10, 15]. In Kox *et al*. study [10], TNF-α, interleukin-6, and interleukin-8 levels were significantly lower in WHM-trained participants, whilst interleukin-10 levels were significantly higher (TNF-α, interleukin-6 and interleukin-8 levels were 53%, 57%, and 51% lower respectively; interleukin-10 levels were 194% higher) compared to non-trained group [10]. Similarly, Zwaag *et al*. endotoxemia study [15], found TNF-α (p = 0.03), interleukin-6 (p = 0.03), and interleukin-8 (p<0.001) levels significantly lower, whereas interleukin-10 levels significantly higher (p = 0.02) in CBR group than CON group. However, similar but less pronounced effects on cytokine levels were found when comparing BRT group to CON group for interleukin-6 (p = 0.04) and interleukin-8 (p = 0.02), but not for interleukin-10 (p = 0.17). Additionally, CEX group did not show any significant changes in TNF-α (p = 0.93), interleukin-6 (p = 0.73), interleukin-8 (p = 0.99), and interleukin-10 (p = 0.44) levels compared to CON [15]. Both studies found similar results although there was a difference in WHBM. Kox *et al*. [10] performed WHBM with retention, while Zwaag *et al*. endotoxemia study [15] performed WHBM without retention.

Inflammation outcomes were also measured in participants with axial spondyloarthritis. During the 8-week WHM training, ESR, median BASDAI, ASDAS-CRP, and serum calprotectin decreased significantly (p = 0.040, p = 0.012, p = 0.044, p = 0.064 respectively) compared to participants not receiving WHM training. However, no statistical significance was found in hs-CRP between both groups (p = 0.103) [7].

### Metabolites response

Zwaag *et al*. [16] showed that lactate and pyruvate play an important role in the anti-inflammatory response in WHM-trained participants. High pyruvate concentration, but not lactate concentration, increased endotoxemia-induced interleukin-10 production, and the combination of both metabolites resulted in an even more prominent and statistically robust increase. Although these two metabolites were highly intercorrelated (p<0.0001), they were not associated with the highly elevated epinephrine levels at any of the examined time points (p-values>0.15). Additionally, pyruvate reduced the production of interleukin-6. Lactate, pyruvate, and the two metabolites combined also tended to reduce endotoxemia-induced TNF-α production; however, significance was not achieved [16]. In Kox *et al*. study [10], lactate levels were significantly higher but not to a clinically relevant level in WHM-trained compared to non-trained participants [10].

### Respiratory parameters

Three papers measured V_E_ [6, 8, 11] and two of them also measured V_T_ and BF [6, 11]. All three papers were investigating whether performing WHBM before an exercise will improve performance. Bahenský *et al*. [6] found the difference in mean values at each load to be significant for V_E_ [6] and Citherlet *et al*. [8] found a significant difference (p = 0.039) between participants performing spontaneous breathing (SB) and WHBM at rest, but the mean difference was not statistically significant after performing an exercise (p>0.05) [8]. Additionally, Marko *et al*. [11] showed that at each load stage, V_E_ depicted statistical insignificance between WHBM and normal breathing (p = 0.138, p = 0.825, p = 0.479, p = 0.489) [11]. Furthermore, for V_T_ and BF measurements, Bahenský *et al*. [6] found the difference in mean values at each load to be significant (p<0.001, p<0.001) [6]; however, Marko *et al*. [11] showed no statistical significance between WHBM participants and normal breathing at any load stage (V_T_: p = 0.630, p = 0.377, p = 0.688, p = 0.087; BF: p = 0.794, p = 0.917, p = 0.956, p = 0.296) [11].

### Blood gas measurements

Two trials examined blood gas measurements [10, 15]. In Kox *et al*. study [10], starting the WHBM resulted in an immediate and profound decrease in pCO_2_ and an increase in pH, but it was not stated if these changes were statistically significant. Furthermore, during WHBM training, the trained group’s oxygen saturation decreased significantly [10]. In contrast, Zwaag *et al*. endotoxemia study [15], found pH and oxygen saturation levels significantly higher in the groups performing WHBM (BRT: p<0.01, p<0.001; CBR: p<0.01, p<0.001 respectively) when compared to CON. Whereas, pCO_2_ was significantly lower in the same groups (BRT: p<0.001; CBR: p<0.001) when compared to CON [15]. In Zwaag *et al*. breathing exercises study [15], oxygen saturation significantly decreased at the end of each retention phase, only when WHBM is performed with retention (p<0.001) compared to without retention. While pH and pCO_2_ were similar in both WHBM exercises, except for a small but statistically significant difference at the final measurement time point (pH: p<0.001; pCO_2_: p<0.001) [15].

### Psychological response

One paper [13] investigated the effect of optimism and neuroticism on WHM. It found that a higher level of expectancy and optimism helped significantly potentiate the effect of WHM while a decrease in neuroticism level was not found to significantly help. Participants in the training group were overall relatively optimistic and low in neuroticism. A higher level of optimism was associated with higher interleukin-10 levels (p<0.05) and epinephrine levels (p<0.01). Neuroticism was not found to be a significant predictor of endotoxin response. Participants’ expectations to overcome the endotoxemia experiment significantly increased from before WHM training to after the endotoxemia experiment (p = 0.003) [13].

Another paper [7] explored the quality of life of participants and found that SF-36 physical and mental component scores significantly increased over the WHM-training period (p = 0.004, p = 0.004 respectively) compared to the non-trained group. While EQ-5D and EQ-5D visual analogue scale did not experience a significant change between the WHM-trained and non-trained groups (EQ-5D: p = 0.933, p = 0.102; EQ-5D visual analogue scale: p = 0.090, p = 0.674) [7].

The last paper [8] reported the results of a customised questionnaire, used for the subjective examination of the three sessions, and Borg RPE scale. Positive effects of increased energy, less fatigue, and improved breathing were reported by 87% of the participants. Almost half of the participants (47%) also said they would think about incorporating the WHBM into their own practices in the future. Additionally, when the experiment was performed using the WHBM, 66.7% of participants rated it the best method in terms of perceived performance compared to 13.3% for SB and 20% for HV. Likewise, 73.3% of participants judged the WHBM to be the best method to perform, while 53.3% judged SB as the worst. However, 33% of the participants using WHBM reported negative effects of deafness and heaviness [8]. Moreover, Borg RPE score was found significantly lower in Bahenský *et al*. (p<0.001) [6] and Citherlet *et al*. (WHBM compared to HV (p = 0.008) and SB (p = 0.017)) [8], meaning that the perceived intensity of a training session is less when WHM is being used [6, 8].

### Reporting of symptoms

Three papers reported experience of flu-like symptoms following endotoxemia administration [10, 13, 15]. Kox *et al*. [10] reported lower flu-like and self-reported symptoms and faster recovery from fever in participants performing WHM [10]. Similarly, Middendorp *et al*. [13] showed that a higher expectation of the training’s effects was associated with lower flu-like clinical symptoms (p<0.01) compared to non-trained group [13]. Likewise, Zwaag *et al*. endotoxemia study [15] results showed that only CEX group had significantly lower flu-like symptoms (p = 0.017) compared to CON group. All other groups had comparable peak symptom scores to CON group (BRT: (p = 0.70); CBR: (p = 0.21)). Additionally, when compared to the CON group, symptoms disappeared considerably faster in all three intervention groups (BRT: p<0.001; CEX: p = 0.01; CBR: p = 0.002) [15]. Overall all participants reported flu-like symptoms but it was significantly lower in participants practising WHM or any one of its pillars such as CEX. The flu-like symptoms also disappeared faster in these participants.

Two papers also reported depressive symptoms [7, 12]. Petraskova Touskova *et al*. [12] found that at the end of the expedition, depressive symptoms were significantly lower in the WHM-trained group compared to non-trained group (p = 0.03), while TSC-40 scores, which measures stess-related symptoms, were higher in the non-trained group, but not significantly [12]. Additionally, Buijze *et al*. [7] found no significant effect on depressive symptoms between WHM-trained and non-trained groups [7].

## Discussion

This is the first systematic review conducted on WHM. The findings of this review suggest that WHM may affect the reduction of inflammation in healthy and non-healthy participants. In addition, there are currently mixed findings on the effect of WHBM and exercise performance. Despite the statistical significance observed in some studies, it must be noted that the quality of the studies is very low, meaning that all the results must be interpreted with caution. Additionally, the low sample size (15–48 individuals per study) and large proportion of males in the studies (86.4%) make the results non-generalizable to the public. Consideration should also be given that participants might experience the placebo effect, where improvements in patients’ symptoms are due to their participation in the therapeutic encounter, with its rituals, symbols, and interactions [27]. For example, *Middendorp et al*. [13] found that a higher level of expectancy and optimism helped significantly potentiate the effect of WHM [13]. However, Zwaag *et al*. [15] who queried the so-called ‘guru effect’ [28] and whether it is necessary to be trained by the creator of the intervention to influence symptomatology [15], found that observed physiological and immunological effects are independent from the individual who provides the WHM treatment intervention.

Out of all the categories, WHM appears to have the most benefit in the stress and pro-inflammatory/anti-inflammatory response categories. Inflammation, especially when chronic, can cause severe complications such as cardiovascular diseases, cancer, diabetes, rheumatoid arthritis, asthma, chronic obstructive pulmonary disease, alzheimer, chronic kidney disease, and inflammatory bowel disease, therefore, reducing inflammation is beneficial [29]. The WHM reduces inflammation using a different mechanism of action (MOA) than other anti-inflammatory interventions. The closest MOA to that of WHM is corticosteroids’ MOA as they both increase interleukin-10 production. WHM increases epinephrine, causing an increase in interleukin-10 which leads to a reduction in pro-inflammatory cytokines [10, 15]. Whereas corticosteroids activate anti-inflammatory genes, including interleukin-10 gene, and inactivate multiple inflammatory genes by inhibiting histone acetyltransferase and recruiting histone deacetylase-2 activity to the inflammatory gene transcriptional complex [30]. The findings of this review suggest that WHM may provide some benefits in healthy and non-healthy people as it was suggested to be safe and might decrease inflammation, unlike corticosteroids, which are only given to non-healthy people due to having many side effects.

WHBM effect on exercise performance category showed mixed results. Although it is too early to decide whether WHBM enhances the performance or not, WHBM could have other potential applications in the physical exertion category. For example, a published letter suggests that WHBM may be helpful in preventing and reversing symptoms of acute mountain sickness (AMS) [31]. Wim Hof and a combination of healthy and non-healthy participants climbed a mountain using the WHBM in 2 days instead of the usual 4–7 days and none of the participants suffered from severe AMS. These findings suggest that a trial on this topic may be of importance, especially for the rescue teams that must ascend quickly [31]. However, although this letter was published in a journal, it was not peer-reviewed and there was no control group; hence the results should be interpreted with caution. Further studies on the effect of WHBM and physical exertion must be conducted before drawing a conclusion.

There are three pillars to WHM: cold exposure, WHBM, and meditation. The latter is a foundation of the other two pillars, thus there were no studies testing it by itself. In this review, cold exposure alone was suggested to have an insignificant effect on epinephrine and cytokine levels [15]. Similarly, the wider literature agrees with this finding. For example, cold water immersion (CWI) did activate the immune system and alter systematic inflammation but not significantly [32–35]. Only one study found CWI helpful in reducing inflammation in rock climbers by reducing vessel permeability toward the site of inflammation [36]. Furthermore, in this review, WHBM alone was claimed to significantly increase epinephrine levels, regardless of the duration of training or trainer, and that increase was more prolonged in participants performing WHBM with retention [15]. On the other hand, cytokine levels were only measured in participants performing WHBM without retention. There were no trials comparing WHBM with retention alone on cytokine levels. When WHBM was performed without retention, pro-inflammatory cytokines significantly decreased, but interleukin-10 levels were not increased significantly [15]. This might be because hypoxia improves interleukin-10 release and reduces the pro-inflammatory response via improved adenosine release [37]. Thus, it would be interesting for future trials to investigate the effects of WHBM with retention alone on cytokine levels. Nevertheless, when cold exposure was combined with WHBM, regardless of whether it was with retention or without, statistical significance was achieved on epinephrine and cytokine levels [10, 15]. Therefore, it is probably best to perform all the pillars of WHM to achieve a significant improvement in immunomodulatory effects. Future trials should investigate whether there is a significant difference between performing WHBM with retention and cold exposure compared to WHBM without retention and cold exposure on epinephrine and cytokine levels.

This review suffers from several limitations. Firstly, only two bibiographic databases were searched to identify suitable papers for this review. Including the database EMBASE, as recommended for inclusion in systematic reviews by Bramer *et al*. [38] may have increased the reach of the search and identified further relevant papers on this topic. Secondly, the search strategy keywords used such as breathing exercise, breathing technique, cold temperature, cold exposure, and cold shower may have excluded studies that used words combined with breathing and cold other than these words. Using breathing and cold as search words alone may have identified further relevant studies. Thirdly, the outcomes were very heterogeneous as many unrelated outcomes are included. This review could have focused on synthesising specific outcomes such as only focusing on outcomes related to stress and inflammation. Fourthly, all the trials had a very high risk of bias. This was due to the lack of a prior published protocol, small sample size, and complexity of blinding the participants and outcome assessors to the intervention. Furthermore, some outcomes such as psychological outcomes were difficult to measure as they are subjective measures usually assessed using a questionnaire. Since the participants were not blinded, it was very difficult to ensure that the answers were honest and valid to the experience. Lastly, the sample size was very small, sometimes affecting the results as any error in measurement or a loss of follow-up can significantly shift the trial results. More evidence needs to be synthesised about WHM before being recommended to the public. An intervention like WHM presents a challenge for rigorous experimental research as participants cannot be blinded to a WHM intervention, therefore the gold standard approach of a double-blinded RCT is not possible. It does appear to offer benefits in attenuating inflammation with minimal serious adverse events and good positive effects of increased energy, less fatigue, and improved breathing [7, 8, 10, 15]. Thus, WHM can probably be used within lifestyle medicine to decrease inflammation in people suffering from inflammatory disorders. However, to be able to recommend WHM in an unwell population, future trials should publish a protocol outlining their experiment before starting the trial, increase the sample size, and make sure to account for loss to follow-up. Additionally, researchers should make sure to blind outcome assessors, and ensure that the outcomes assessed are objective and not subjective.

## Conclusion

Considering all the studies, the WHM may produce promising immunomodulatory effects but more research of higher quality is needed to substantiate this finding. The combination of cold exposure and WHBM appeared to most effectively reduce the inflammatory response. Hence, all the pillars of the WHM are important to extract the benefits. The focus of future studies should further investigate the benefits of WHM in preventing or treating diseases, such as inflammatory disorders, in non-healthy participants and explore the use of WHBM in enhancing exercise performance. Studies about WHM have not yet investigated all the beneficial claims the WHM states to have. Future studies may provide valuable insights about WHM as there is still much to explore.

## Supporting information

S1 Checklist PRISMA 2009 checklist. (DOC)

S1 Table SIGN tool assessment. (PDF)
