# Supplementary material for: Does the Wim Hof Method have a beneficial impact on physiological and psychological outcomes in healthy and non-healthy participants? A systematic review
Source: PLoS One. 2024 Mar 13;19(3):e0286933. doi: 10.1371/journal.pone.0286933 (PMC10936795; doi:10.1371/journal.pone.0286933)
Supplement: S1 Table — (PDF) [file pone.0286933.s002.pdf]

S1 Table. SIGN tool assessment.

	Petraskova Touskova *et al.*, 2022
1.1 The study addresses an appropriate and clearly focused question.	Yes
1.2 The two groups being studied are selected from source populations that are comparable in all respects other than the factor under investigation.	Yes
1.3 The study indicates how many of the people asked to take part did so, in each of the groups being studied.	No
1.4 The likelihood that some eligible subjects might have the outcome at the time of enrolment is assessed and taken into account in the analysis.	Does not apply
1.6 Comparison is made between full participants and those lost to follow up, by exposure status.	No
1.7 The outcomes are clearly defined.	Yes
1.8 The assessment of outcome is made blind to exposure status. If the study is retrospective this may not be applicable.	No
1.9 Where blinding was not possible, there is some recognition that knowledge of exposure status could have influenced the assessment of outcome.	Can’t say
1.10 The method of assessment of exposure is reliable.	Can’t say
1.11 Evidence from other sources is used to demonstrate that the method of outcome assessment is valid and reliable.	Yes

1.12 Exposure level or prognostic factor is assessed more than once.	Does not apply
1.13 The main potential confounders are identified and taken into account in the design and analysis.	No
1.14 Have confidence intervals been provided?	No
2.1 How well was the study done to minimise the risk of bias or confounding?	Unacceptable – reject 0
2.2 Taking into account clinical considerations, your evaluation of the methodology used, and the statistical power of the study, do you think there is clear evidence of an association between exposure and outcome?	Can't say
2.3 Are the results of this study directly applicable to the patient group targeted in this guideline?	No
